# When “CAM” is not CAM: Interpreting national cancer database analyses in breast cancer

**DOI:** 10.1093/oncolo/oyag187

**Published:** 2026-05-14

**Authors:** Michael Jeitler, Holger Cramer

**Affiliations:** Institute of General Practice and Interprofessional Care, University Hospital Tuebingen, Tuebingen 72076, Germany; Robert Bosch Center for Integrative Medicine and Health, Bosch Health Campus, Stuttgart 70376, Germany; Institute of Social Medicine, Epidemiology and Health Economics, Charité—Universitätsmedizin Berlin, Corporate Member of Freie Universität Berlin and Humboldt-Universität zu Berlin, Berlin 10117, Germany; Institute of General Practice and Interprofessional Care, University Hospital Tuebingen, Tuebingen 72076, Germany; Robert Bosch Center for Integrative Medicine and Health, Bosch Health Campus, Stuttgart 70376, Germany

**Keywords:** breast cancer, complementary and alternative medicine, integrative oncology, National Cancer Database, survival, treatment patterns, construct validity, undertreatment

Complementary and alternative medicine (CAM) remains a challenging topic in oncology. Many patients seek supportive strategies during and after active treatment, often to reduce symptom burden, improve quality of life, or regain a sense of agency during the cancer journey. At the same time, oncologists are rightly concerned about treatment delay, substitution of evidence-based care, and potentially harmful nonconventional practices. Against this background, studies addressing CAM use in cancer are of considerable clinical and public interest. That interest, however, also means that terminological precision is essential. The central interpretive issue is therefore not only terminology but whether the exposure being studied validly represents the clinical construct readers understand as CAM or integrative oncology.

A recent analysis by Ayoade et al. illustrates this challenge clearly. Using National Cancer Database data, the authors examined associations of CAM with treatment patterns and survival in breast cancer and concluded that CAM used instead of traditional therapies was associated with reduced survival.^1^ Their analysis addresses a clinically important concern. However, the operational definition underlying the exposure is much narrower than the article’s general framing may suggest: the analysis does not study CAM as commonly encountered in oncology practice but a very small registry-coded subgroup defined by receipt of treatment administered by nonmedical personnel.

A recurring problem in database-based analyses is that the exposure labeled as “CAM” may differ substantially from what clinicians, patients, and readers usually understand by that term. In clinical practice and supportive cancer care research, CAM often refers to a broad range of complementary approaches used alongside conventional treatment. By contrast, database variables may capture a much narrower phenomenon, such as documented nonmedical or alternative treatment behavior. If these constructs are presented under the same label, the resulting findings may be interpreted more broadly than the data can support. This is therefore an exposure-definition and construct-validity problem, not merely a semantic concern.

In the study by Ayoade et al., CAM is based on the National Cancer Database designation of treatment administered by nonmedical personnel. At the same time, the article discusses CAM in broader terms and refers to approaches such as acupuncture, massage, mindfulness-based practices, and integrative oncology. Yet these approaches are often used as supportive adjuncts rather than as alternatives to cancer-directed treatment.^1^ The study therefore appears to capture a narrow subgroup with documented nonmedical alternative cancer treatment rather than the broader use of complementary therapies for supportive care.

This distinction matters because supportive and integrative approaches are already addressed in formal guideline work. Integrative oncology has been described as an evidence-informed, patient-centered field that combines selected complementary interventions with conventional care, and guideline documents have addressed supportive integrative therapies in breast cancer symptom management.^2^^,^^3^ This distinction is also consistent with the current German S3 guideline on complementary medicine in oncology, which approaches complementary interventions within an evidence-based oncologic framework rather than as a substitute for standard cancer treatment.^4^

The very low observed prevalence further reinforces this concern. Among more than 2.15 million women included in the analysis, only 273 were classified as receiving CAM alone and 568 as receiving both CAM and traditional therapy.^1^ This is substantially lower than prevalence estimates from broader survey-based studies of complementary health approach use among people with cancer. In a nationally representative analysis, 35.3% of adults with a recent cancer diagnosis reported complementary health approach use, and 43.6% of those with breast cancer reported such use, whereas only 2.3% reported use specifically for cancer treatment.^5^ These data suggest that broad complementary use and the much narrower subgroup pursuing nonconventional approaches specifically as cancer treatment should not be treated as interchangeable constructs. The authors themselves acknowledge that the National Cancer Database likely undercaptures CAM use and suggest that some patients using CAM may even have been included in the no-treatment group.^1^ If so, the comparison may reflect a highly selected subgroup with documented alternative-treatment coding rather than a comparison between women who do and do not use CAM in the broader clinical sense.

A second issue concerns treatment timing. The authors appropriately recognize the potential for immortal time bias because the CAM component tended to start later than other treatments, and they present a landmarked, unadjusted analysis in the Supplement.^1^ However, the main adjusted results are derived from a Cox proportional hazards model, and the paper does not clearly explain whether differential treatment timing was handled in that adjusted analysis through landmarking or time-dependent exposure modeling.^1^ Because the adjusted hazard ratios are central to the interpretation of the study, this point is not merely technical. In database-based analyses, where exposures may accrue after diagnosis and after initiation of standard therapy, time handling can materially affect apparent associations.

The interpretation of the combination cohort also deserves caution. The subgroup receiving both CAM and traditional therapy was small, and the authors explicitly note limited power for head-to-head subgroup comparisons.^1^ They also state that they could not isolate patients who completed conventional treatment and only then added CAM.^1^ This is critical because worse outcomes in this cohort may be entangled with less complete receipt of standard oncologic therapy. Accordingly, the observed association in that cohort may reflect a heterogeneous mixture of care pathways, including undertreatment, treatment substitution, delayed initiation, selective omission of standard modalities, or incomplete adherence to treatment recommendations. It should therefore not be conflated with adjunctive integrative care as commonly understood in oncology practice.

Residual confounding further limits interpretation. Although the adjusted model includes demographic, socioeconomic, health-system, comorbidity, year-of-diagnosis, stage, and income variables, it does not appear to include key breast cancer-specific clinical factors such as receptor status, HER2 status, tumor grade, tumor biology, recurrence risk, or finer-grained treatment indication.^1^ These variables strongly influence both prognosis and treatment choice. In addition, stage information was missing for a notable proportion of patients in table 1, including 34.4% of CAM-only patients, 35.4% of the combination cohort, 43.7% of the traditional-therapy cohort, and 42.5% of the no-treatment cohort.^1^ Given the central role of stage in breast cancer prognosis and treatment selection, this degree of missingness weakens confidence in adjustment and subgroup interpretation.

These concerns are not entirely new. Similar issues were raised after the earlier National Cancer Database analysis by Johnson et al., which was also criticized for using a narrow operationalization of “complementary medicine” while being interpreted more broadly in clinical and public discourse.^6^^,^^7^ The broader lesson from both papers is not that concerns about replacement of standard oncologic care are misplaced. They are not. Rather, the lesson is that studies of this kind require especially careful terminology, because the clinical, communicative, and public implications of the term “CAM” extend well beyond the subset of patients captured by such registry coding.

The article also appears to contain internal reporting inconsistencies that may merit correction.^1^ More importantly, however, it highlights a broader methodological issue: database-based studies may identify clinically important patterns of alternative treatment behavior, but that does not necessarily mean they capture CAM or integrative oncology as those terms are commonly used in contemporary cancer care.

For oncology clinicians, this distinction is not semantic. It shapes how evidence is communicated to patients, how supportive care is framed, and how the public interprets the role of complementary approaches in cancer treatment. Studies addressing treatment substitution deserve attention. But their findings should be described with terms that accurately reflect the underlying exposure. Greater terminological precision would help prevent overgeneralization and support more balanced communication about supportive and integrative cancer care in oncology. Future analyses using this National Cancer Database variable would therefore be more accurately framed as studies of “registry-coded nonmedical alternative cancer treatment” or “documented alternative-treatment behavior,” rather than as studies of CAM or integrative oncology as a whole.
